# Supplementary material for: mTOR Inhibition via Rapamycin Treatment Partially Reverts the Deficit in Energy Metabolism Caused by FH Loss in RPE Cells
Source: Antioxidants (Basel). 2021 Dec 3;10(12):1944. doi: 10.3390/antiox10121944 (PMC8750186; doi:10.3390/antiox10121944)
Supplement: Supplementary file 1 [file antioxidants-10-01944-s001.zip › antioxidants-1462966-supplementary.pdf]

antioxidants

MDPI

Research Article

Supplementary material: mTOR inhibition *via* Rapamycin treatment partially reverts the deficit in energy metabolism caused by FH loss in RPE cells

David A. Merle[1,2], Francesca Provenzano[3], Mohamed Ali Jarboui[1], Ellen Kilger[1], Simon J. Clark[1,4,5]; Michela Deleidi[3], Angela Armento[1*] and Marius Ueffing[1,3*]

[1] Institute for Ophthalmic Research, Department for Ophthalmology, Eberhard Karls University of Tübingen, Tübingen, Baden-Württemberg, 72076, Germany

angela.armento@uni-tuebingen.de (A.A.), mohamed-ali.jarboui@uni-tuebingen.de (M.A.J.), ellen.kilger@uni-tuebingen.de (E.K.), marius.ueffing@uni-tuebingen.de (M.Ue.)

[2] Department of Ophthalmology, Medical University of Graz, 8036 Graz, Austria. david.merle@medunigraz.at (D.A.M.)

[3] German Center for Neurodegenerative Diseases (DZNE), Tübingen, Baden-Württemberg, 72076, Germany. Michela.Deleidi@dzne.de (M.D.), Francesca.Provenzano@dzne.de (F.P.)

[4] University Eye Clinic, Department for Ophthalmology, Eberhard Karls University of Tübingen, Tübingen, Baden-Württemberg, 72076, Germany

simon.clark@uni-tuebingen.de (S.J.C.)

[5] Lydia Becker Institute of Immunology and Inflammation, Faculty of Biology, Medicine and Health, University of Manchester, Manchester, M13 9PT, UK

* Both authors share equal last authorship (A.A., M.Ue.); correspondence should be addressed to:

Angela Armento, PhD, angela.armento@uni-tuebingen.de, Phone: +49 7071 29 84953; Marius Ueffing, PhD, marius.ueffing@uni-tuebingen.de. Institute for Ophthalmic Research, Department for Ophthalmology, Eberhard Karls University of Tübingen, Tübingen, Baden-Württemberg, 72076, Germany

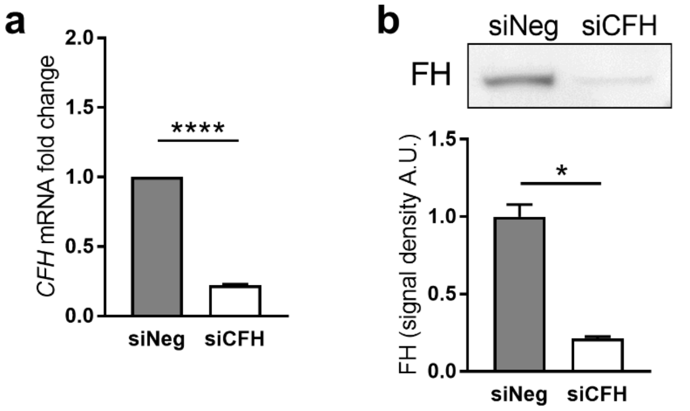

Figure S1: Efficiency of CFH silencing in RPE cells. hTERT-RPE1 cells were silenced for 24 hours with negative control (siNeg) or CFH specific (siCFH) siRNA and then incubated for 48 hours. Cell pellets and cell culture supernatants were collected for RNAand protein extraction. a) Evaluation of *CFH* expression by qRT-PCR analyses. Data are normalized to the housekeeping gene PRLP0 using Δ ΔCt methods. SEM is shown, n=4. b) Western blot analyses of FH protein levels in cell culture supernatants of hTERT-RPE1 cells. Quantification of signal density of 3 independent experiments is shown Significance was assessed by Student's t-test.

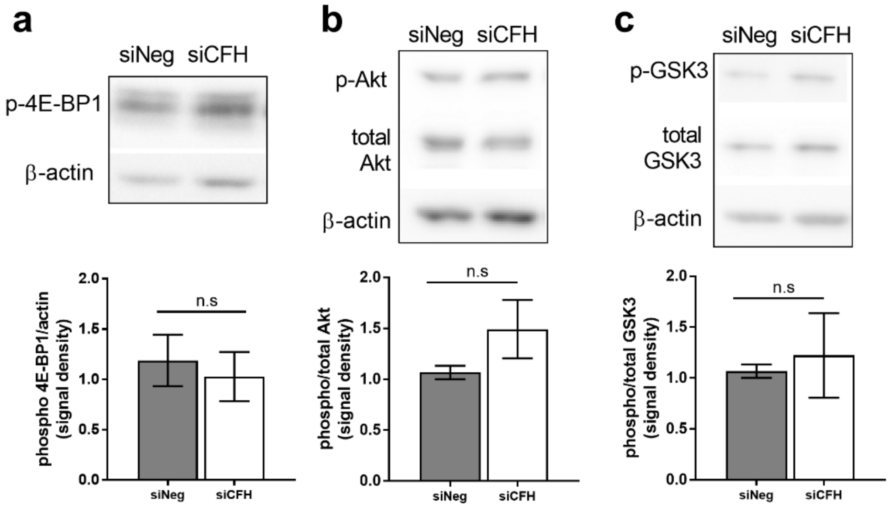

Figure S2: Effects of FH loss on signaling pathways. hTERT-RPE1 cells were silenced for 24 hours with negative control (siNeg) or CFH specific (siCFH) siRNA and then incubated for 48 hours. Cell pellets were collected for protein extraction. Western blot analyses of: a)phosphorylated levels of 4E-BP1; b) phosphorylated and total levels of Akt; c) phosphorylated and total levels of GSK3. Total β-actin was used as loading control and quantification of the signal intensity is shown. N=3. SEM is shown.

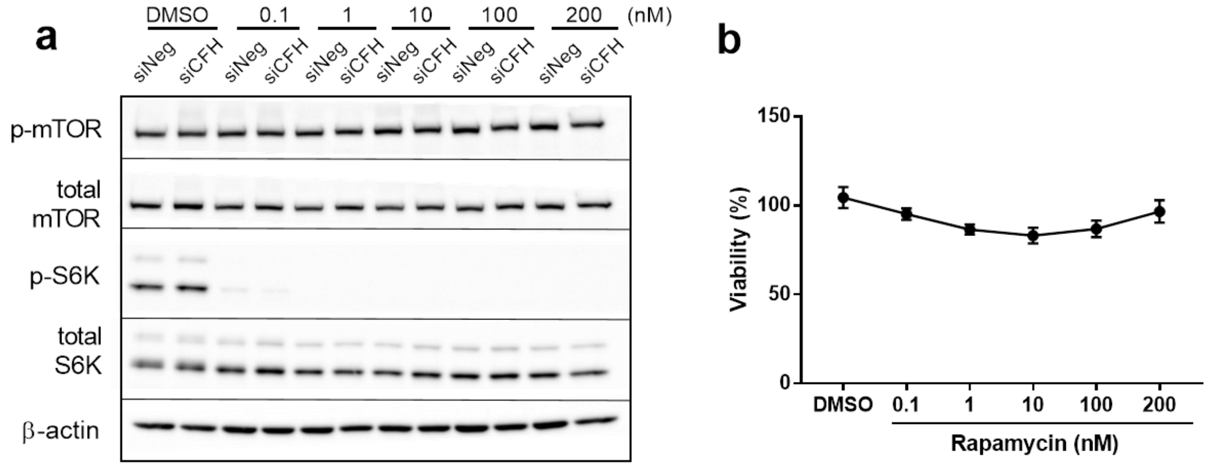

Figure S3: effects of Rapamycin concentration on mTOR pathway and viability. hTERT-RPE1 cells were silenced for 24 hours with negative control (siNeg) or CFH specific (siCFH) siRNA and then incubated for 48 hours with increasing concentrations of rapamycin as indicated. a) Cell pellets were collected for protein extraction. Western blot analyses of phosphorylated and total levels of mTOR; phosphorylated and total levels of S6K. Total β-actin was used as loading control. b) Viability assessed *via* MTT assay. N=3. SEM is shown.

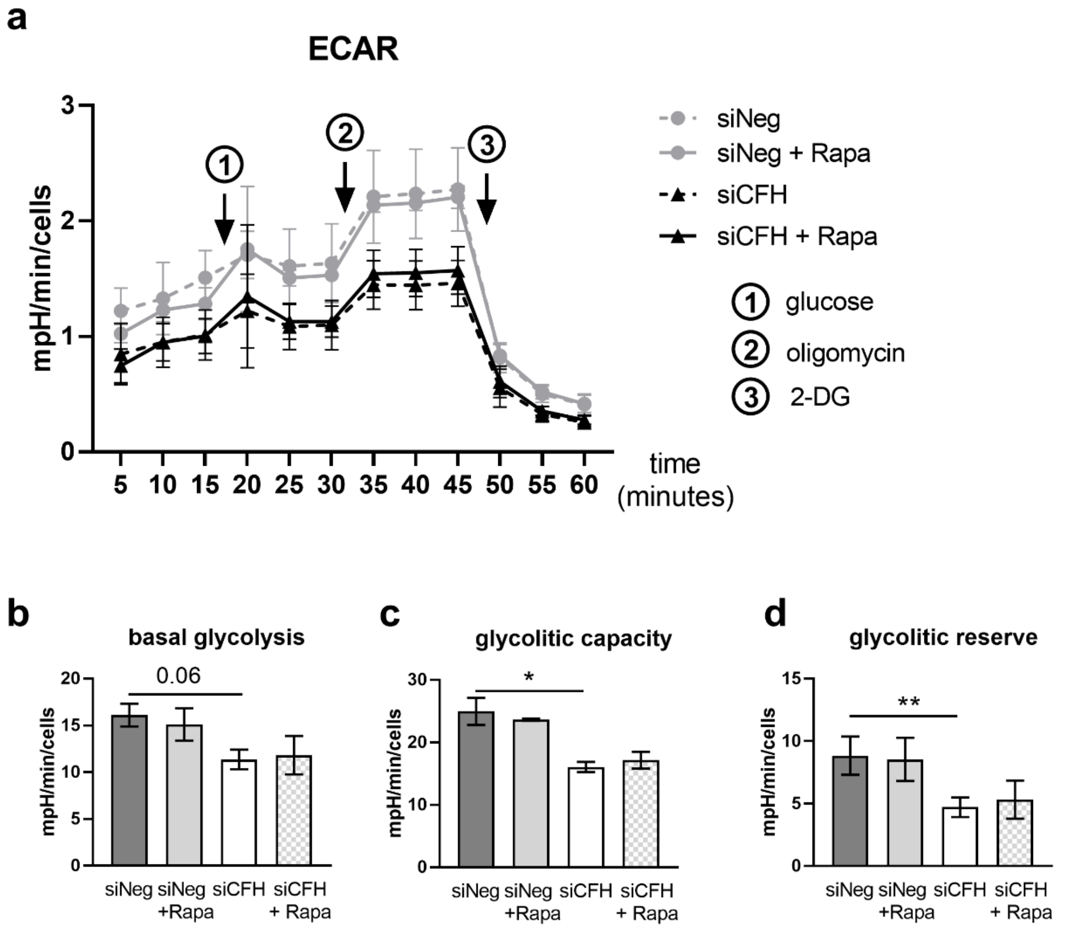

Figure S4: Effects of rapamycin on the glycolysis of RPE cells. (a) hTERT-RPE1 cells were silenced for 24 hours with negative control (siNeg) or CFH specific (siCFH) siRNA, seeded on seahorse plates and incubated in SFM containing 200nM rapamycin or DMSO. Curves represent the extracellular acidification rates (ECAR) measured after 48 hours. SEM shown, n = 5. Arrows indicate injection of glucose (1), oligomycin (2), 2-DG (3). (b–d) Parameters of glycolysis were calculated based on the data shown in (a). (b) Basal glycolysis, (c) glycolytic capacity, (d) glycolytic reserve. Significance was assessed by Student's t-test. $^{*}p < 0.05$, $^{**}p < 0.01$.

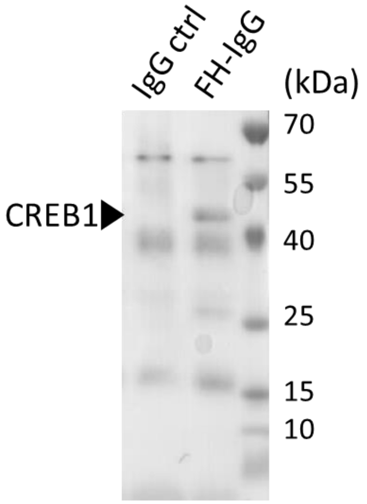

Figure S5: *Western blot for CREB1 using eluates from FH-IPs and IgG control-IPs.* hTERT-RPE1 cells were cultured for 48h. Cell pellets were collected for protein extraction and Immunoprecipitation was performed using a FH antibody (FH-IgG) or an unspecific IgG as a control (IgG ctrl). After elution and methanol/chlorofrom precipitation, a small aliqout was kept for western blotting, while the rest of the samples were processed for massspectrometric (MS) analysis. As CREB1 was among the top hits in the FH interactome MS-dataset, we tested the eluate aliquots for presence or absence of CREB1 using a well-validated, commercial antibody (Cat No. 12208-1-AP, Proteintech). CREB1 was detected in FH IgG samples, while IgG controls did not give a CREB1-specific signal. The non-indicated bands in both samples result from crossreactivity with the antibodies used for the IP.

Table S1: Significantly enriched proteins in FH samples. The whole dataset including LFQ intensities and additional parameters can be retrieved from ProteomeXchange.

Gene names	Majority protein IDs	p-Value	Mean Difference CFH vs Ctrl
**LEPRE1**	Q32P28	1.6235E-14	23.7274313
**CCDC136**	Q96JN2	3.33475E-14	26.80236149
**CREB1;ATF1**	P16220;P18846	4.99625E-14	24.20196581
**BRAP**	Q7Z569	6.50194E-14	24.82372427
**RABGGTB**	P53611	1.05303E-13	22.96165276
**SPTB**	P11277	1.07642E-13	24.1661644
**FAM184B**	Q9ULE4	1.10747E-13	29.97203302
**E2F4**	Q16254	1.12392E-13	23.22352886
**UCN3**	Q969E3	1.34454E-13	23.88911533
**RAD17**	O75943	4.11222E-13	25.5679965
**NCKAP5L**	Q9HCH0	9.4782E-13	21.06704617
**ITCH**	Q96J02	1.9754E-12	23.4377985
**ATAD3A**	Q9NVI7	4.30175E-12	21.26448727
**LUC7L3**	O95232	5.21921E-12	21.21871471
**KIAA1107**	Q9UPP5	7.15949E-12	20.27953434
**GNB4**	Q9HAV0	8.33428E-12	20.82388973
**CACTIN**	Q8WUQ7	8.67736E-12	21.57487106
**SPATS2L**	Q9NUQ6	1.44826E-11	20.8269186
**CXorf56**	Q9H5V9	1.79518E-11	22.04429579
**ATAD3B**	Q5T9A4	2.50029E-11	24.08491898
**PSMB3**	P49720	2.57109E-11	19.10311842
**TFDP2**	Q14188	2.68949E-11	21.74063826
**NARS2**	Q96I59	3.21775E-11	22.99020576
**TFDP1**	Q14186	4.03805E-11	23.44895029
**ARHGEF40**	Q8TER5	6.48736E-11	18.54688215
**DST**	Q03001	6.67033E-11	23.83796215
**DYNC1I2**	Q13409	1.16082E-10	18.75141859
**ALOX12B**	O75342	1.22401E-10	19.90516663
**CAMTA2**	O94983	1.51748E-10	18.84609842
**TCEANC2**	Q96MN5	1.61163E-10	22.30599213
**MICAL2**	O94851	1.91471E-10	22.78196573
**LUC7L2**	Q9Y383	2.64243E-10	19.90857267
**NALCN**	Q8IZF0	3.9284E-10	23.65266705
**HSD17B12**	Q53GQ0	5.14354E-10	19.76246071
**G3BP2**	Q9UN86	5.34248E-10	20.74985456
**BMP1**	P13497	6.01063E-10	17.74047995
**MCM5**	P33992	6.15221E-10	21.86290598

**MTCL1**	Q9Y4B5	8.72964E-10	19.16291666
**GTF2F1**	P35269	1.40001E-09	21.96182203
**ARL6IP4**	Q66PJ3	2.82612E-09	20.32786083
**ASB1**	Q9Y576	3.13137E-09	20.32534218
**FHOD1**	Q9Y613	4.61619E-09	21.79634285
**WRNIP1**	Q96S55	9.89487E-09	20.77000856
**C1QBP**	Q07021	1.38315E-08	22.04387093
**FARSB**	Q9NSD9	2.03539E-08	21.6620307
**MCM3**	P25205	3.04929E-08	21.55954313
**RAI14**	Q9P0K7	5.289E-08	22.09490585
**TJP1**	Q07157	9.6431E-08	9.060780525
**PRDX2**	P32119	9.48772E-07	2.160865784
**VWA8**	A3KMH1	2.58649E-06	8.374697685
**IGHG2**	P01859	2.87324E-06	2.267016888
**RB1**	P06400	1.69138E-05	5.728475571
**E2F3**	O00716	0.000123596	4.44920063
**HS2ST1**	Q7LGA3	0.000805649	2.787140369
**HDLBP**	Q00341	0.001226053	16.58484364
**MYO1C**	O00159	0.00368581	1.961327553
**IGHG1**	P0DOX5;P01857	0.004173774	1.447899342
**C9orf43**	Q8TAL5	0.005461557	19.79761076
**METTL15**	A6NJ78	0.006076881	18.81872368
**SYNE2**	Q8WXH0	0.006637627	19.43278456
**GTF3C1**	Q12789	0.007003875	15.98174143
**GTF3C5**	Q9Y5Q8	0.008248568	17.46328402
**MAP1A**	P78559	0.008712723	15.73902512
**PUF60**	Q9UHX1	0.0089359	16.43379593
**SLAIN2**	Q9P270	0.009466481	16.88302183
**LRSAM1**	Q6UWE0	0.009942525	17.61318159
**PLOD2**	O00469	0.009959096	15.37081671
**CCAR1**	Q8IX12	0.010096252	16.58990526
**GTPBP2**	Q9BX10	0.010515084	15.82120132
**LCN1;LCN1P1**	P31025;Q5VSP4	0.010799184	16.08209801
**SH3D19**	Q5HYK7	0.011342001	16.60372448
**LOR**	P23490	0.011838207	15.43536043
**FARSA**	Q9Y285	0.012004423	16.31003904
**GTF3C6**	Q969F1	0.012004478	16.02385902
**MYO9A**	B2RTY4	0.012006823	16.52427387
**DDX39B;DDX39A**	Q13838;O00148	0.012006966	14.40969276
**MGA**	Q8IWI9	0.012008256	15.91280937
**PSMD13**	Q9UNM6	0.012008775	14.24829388
**PSMB6**	P28072	0.012009292	16.03938675

**SH2D3C**	Q8N5H7	0.012009504	15.85227156
**RAB1A;RAB1B;RAB1C;RAB8B;RAB8A;RAB10;RAB13;RAB15**	P62820;Q9H0U4;Q92928;Q92930;P61006;P61026;P51153;P59190	0.012009763	16.35257006
**SLC39A7**	Q92504	0.012013979	13.94072628
**SRP9**	P49458	0.012014413	15.26320553
**WDR1**	O75083	0.012015373	14.04478264
**MANBA**	O00462	0.012015711	15.70327425
**PSMA3**	P25788	0.012016117	15.26424742
**PDIA6**	Q15084	0.012018752	16.17472506
**TSEN15**	Q8WW01	0.012019648	15.62000895
**INTS1**	Q8N201	0.012029182	16.50816965
**ANKRD12**	Q6UB98	0.012034191	16.93458366
**TMEM33**	P57088	0.012037901	16.90490389
**LRP8**	Q14114	0.012045052	14.9916234
**PTPRG**	P23470	0.012062199	17.77592945
**PFKP**	Q01813	0.012066504	16.19368744
**PSME3**	P61289	0.012072013	17.23930597
**PRPF4B**	Q13523	0.012073328	17.01796865
**QPCTL**	Q9NXS2	0.012084156	15.13104677
**TSEN2**	Q8NCE0	0.01209207	16.30651617
**SMC3**	Q9UQE7	0.01210472	14.55299091
**SEPTIN9**	Q9UHD8	0.012117349	14.87176657
